# Identifying groups of people with similar sociobehavioural characteristics in Malawi to inform HIV interventions: a latent class analysis

**DOI:** 10.1002/jia2.25615

**Published:** 2020-09-28

**Authors:** Aziza Merzouki, Amanda Styles, Janne Estill, Erol Orel, Zofia Baranczuk, Karen Petrie, Olivia Keiser

**Affiliations:** ^1^ Institute of Global Health University of Geneva Geneva Switzerland; ^2^ University of Dundee Dundee United Kingdom; ^3^ Institute of Mathematical Statistics and Actuarial Science University of Bern Bern Switzerland; ^4^ Department of Psychology University of Zurich Zurich Switzerland; ^5^ Institute of Mathematics University of Zurich Zurich Switzerland

**Keywords:** latent class analysis, HIV prevalence, HIV testing, risk groups, spatial distribution, Malawi

## Abstract

**Introduction:**

Within many sub‐Saharan African countries including Malawi, HIV prevalence varies widely between regions. This variability may be related to the distribution of population groups with specific sociobehavioural characteristics that influence the transmission of HIV and the uptake of prevention. In this study, we intended to identify groups of people in Malawi with similar risk profiles.

**Methods:**

We used data from the Demographic and Health Survey in Malawi (2015 to 2016), and stratified the analysis by sex. We considered demographic, socio‐behavioural and HIV‐related variables. Using Latent Class Analysis (LCA), we identified groups of people sharing common sociobehavioural characteristics. The optimal number of classes (groups) was selected based on the Bayesian information criterion. We compared the proportions of individuals belonging to the different groups across the three regions and 28 districts of Malawi.

**Results:**

We found nine groups of women and six groups of men. Most women in the groups with highest risk of being HIV positive were living in female‐headed households and were formerly married or in a union. Among men, older men had the highest risk of being HIV positive, followed by young (20 to 25) single men. Generally, low HIV testing uptake correlated with lower risk of having HIV. However, rural adolescent girls had a low probability of being tested (48.7%) despite a relatively high HIV prevalence. Urban districts and the Southern region had a higher percentage of high‐prevalence and less tested groups of individuals than other areas.

**Conclusions:**

LCA is an efficient method to find groups of people sharing common HIV risk profiles, identify particularly vulnerable sub‐populations, and plan targeted interventions focusing on these groups. Tailored support, prevention and HIV testing programmes should focus particularly on female household heads, adolescent girls living in rural areas, older married men and young men who have never been married.

## 1 INTRODUCTION

Diagnosis of HIV‐positive people is a critical step to improve access to treatment and control the HIV epidemic. In Eastern and Southern Africa, where HIV prevalence is the highest in the world [1], the proportion of people living with HIV who know their status increased from 77% in 2015 to 85% in 2018 [1]. In particular, Malawi has made good progress towards reaching the 90‐90‐90 UNAIDS target. In 2018, an estimated 90% of HIV‐positive persons knew their status, 87% of those were on ART, and 89% of persons on ART were virally suppressed [1]. Key national policies include active index testing, HIV self‐testing, ART optimization and annual viral load testing in combination with a data monitoring system to track the effectiveness of interventions [2]. Despite this progress, disparities between region and populations remain and interventions that better target those who are most at risk are needed to control the epidemic.

The disparities between regions may result from the spatial distribution of population groups with specific sociobehavioural characteristics that promote or impede the transmission of HIV and the uptake of preventive measures. Improving our understanding of the characteristics of different groups in the population, their risk of being HIV positive and their uptake of HIV testing is essential to design more targeted prevention and care programmes. Previous studies have shown that clustering analysis allows to identify similarity patterns and find hidden sub‐groups of people with potentially different risk levels and drivers of having or acquiring HIV [3]. When comparing sub‐Saharan African (SSA) countries, characteristic sociobehavioural profiles of the populations were found to coincide with the level of HIV prevalence and incidence in the countries [4]. Within certain SSA countries, high‐risk groups have been studied, but the studies have mainly focused on women [5, 6, 7] and the spatial distribution of the identified groups across the country has not been examined. One recent analysis mapped the proportion of high‐risk men and women across Malawi to study whether their distribution could explain the spatial variability of HIV prevalence in the country, but the definition of high‐risk behaviour was based only on the total number of lifetime sexual partners of individuals [8].

In this study, we aimed to identify different sociobehavioural groups of women and men in Malawi and to assess their risk of being HIV positive. We used Latent Class Analysis (LCA) to identify population groups with different sociobehavioural characteristics, based on the latest Demographic and Health Survey (DHS) [9]. We also compared the most common groups across regions and districts of the country.

## 2 METHODS

### 2.1 Data

We used data from the latest available DHS (2015 to 2016) for Malawi (details in Supplementary Material). The DHS programme collects nationally representative data and covers a wide range of indicators for population and health. Overall 24 562 women aged 15 to 49 years in 26 361 selected households and 7478 men aged 15 to 54 in one‐third of the selected households were interviewed; corresponding to a response rate of 98% in women and 95% in men [10]. For a representative subset of persons also HIV testing is done. Of the 8497 women and 7542 men aged 15 to 49 eligible for testing, 93% of women and 87% of men provided specimens for HIV testing [10, 11]. The sub‐samples of persons with known HIV‐status have been weighted using the HIV‐test sample weight. Details on HIV testing are provided in the Supplementary Material.

Two researchers (AM and OK) preselected the variables based on the framework by Boerma & Weir [12], a prior non‐systematic literature search, and some practical constraints (i.e. amount of missing values, collinearity between variables). The following variables were considered: place of residence (rural; urban), age (≤19; 20 to 25; 26 to 35; >35 years), literacy (can read a complete sentence: yes; no), access to media at least once a week (yes; no), marital status (formerly married or living in a union; never married nor lived in a union), sex of household head (female; male), age at first sexual intercourse (<16; 16 to 18; 19 to 21; >21), condom use (wife justified to ask husband to use a condom when he has a sexually transmitted infection (STI); not justified), wife beating (at least one reason justifies wife beating; no reason justifies it), comprehensive correct knowledge about AIDS (yes; no) and prior HIV testing (ever been tested; never tested). We also considered the district and region of residence of respondents for mapping. The exact definition of each variable is provided in the DHS guide [13].

### 2.2 Analysis

We conducted separate analyses for women and men. Since our study focused on sexual transmission of HIV, we excluded persons who never had sexual intercourse. We used multiple imputation by chained equations (MICE) [14, 15] to complete missing data (Supplementary Material). Some implausible or inconsistent values were set to missing before imputation.

We identified groups of people sharing common sociobehavioural characteristics using LCA [16], a multivariate technique that groups individuals with similar characteristics in latent (i.e. not directly observable) classes (groups). We used maximum likelihood estimation to calculate the probability of an individual belonging to a group. Individuals were grouped into the class for which they had the highest membership probability. We selected the number of groups that minimized the Bayesian information criterion (BIC) [17]. We performed the final analysis based on a subset of variables identified as the most important for clustering (Supplementary Material). We selected these variables for men and women separately based on the variance of their categories’ probabilities across the groups, similarly to the method by Dean and Raftery [18]. Reducing the number of variables allows us to reduce the number of groups, thus facilitating interpretability.

We analysed the HIV prevalence and the self‐reported prior HIV testing uptake in each LCA group. We defined HIV prevalence as the proportion of positive tests among the positive and negative test results. We also analysed the distribution of the groups across regions and districts, and assessed whether it could help to explain the wide spatial variability of HIV prevalence.

We conducted the analysis using the open source R language, version 3.5.1. We used the implementation of LCA provided in poLCA package, version 1.4.1 [19, 20]. The code is available on GitLab (https://gitlab.com/AzizaM/dhs_malawi_lca).

### 2.3 Ethics approval

Data from the DHS programme are freely available on request. Details on the ethical review process of DHS are available on the programme’s website https://www.dhsprogram.com/What‐We‐Do/Protecting‐the‐Privacy‐of‐DHS‐Survey‐Respondents.cfm.

## 3 RESULTS

The survey included 24 562 women and 7478 men. After imputing incomplete data and removing respondents who never had sexual intercourse, we performed the analysis on 21 564 women and 6379 men. The HIV status was known for 6750 women (5889 HIV negative; 861 HIV positive) and 5642 men (5168 HIV negative; 474 HIV positive).

### 3.1 Women’s groups

We found an optimal number of nine groups for women based on seven variables: age, literacy, marital status, location of residence, sex of household head, having ever been tested for HIV and employment (Table 1). Entropy statistic and average posterior probabilities from LCA for women are provided in Table S2. Two groups had a high HIV prevalence (*Groups 2* and *8*). *Group 8* had an HIV prevalence of 37.2%, and was predominantly urban (100.0%) and literate (87.8%). *Group 2* had a prevalence of 21.2%, and was predominantly rural (96.6%). In comparison, other groups had a median HIV prevalence of 10.0% (IQR 7.9% to 12.3%). *Groups 2* and *8* both had high proportions of female‐headed households (100.0% and 83.2% respectively) and most women had lived in a union before (76.2% and 59.1% respectively).

**Table 1:** Groups of women based on the seven most important variables: age, literacy, marital status, location of residence, sex of household head, having ever been tested for HIV and employment. Data from 21 564 women were analysed

Group	1	2	3	4	5	6	7	8	9
Age									
19 and under	1044 (77.8%)	85 (2.7%)	121 (2.1%)	687 (13.5%)	77 (7.8%)	53 (1.8%)	229 (24.2%)	0 (0.0%)	154 (19.4%)
20 to 25	267 (19.9%)	477 (15.2%)	1103 (19.4%)	2157 (42.4%)	410 (41.6%)	620 (20.9%)	592 (62.4%)	0 (0.0%)	151 (19.0%)
26 to 35	31 (2.3%)	1119 (35.8%)	2019 (35.5%)	2075 (40.7%)	403 (40.9%)	1544 (52.0%)	127 (13.4%)	314 (52.0%)	121 (15.2%)
Over 35	0 (0.0%)	1447 (46.3%)	2452 (43.1%)	174 (3.4%)	97 (9.8%)	752 (25.3%)	0 (0.0%)	290 (48.0%)	371 (46.5%)
Literacy									
Good reader^a^	986 (73.4%)	1452 (46.4%)	1708 (30.0%)	3900 (76.6%)	799 (81.0%)	2970 (100.0%)	936 (98.7%)	530 (87.8%)	235 (29.4%)
Poor reader^b^	357 (26.6%)	1675 (53.6%)	3987 (70.0%)	1193 (23.4%)	188 (19.0%)	0 (0.0%)	12 (1.3%)	74 (12.2%)	562 (70.6%)
Current marital status									
Formerly married or lived in a union	41 (3.0%)	2384 (76.2%)	60 (1.1%)	237 (4.7%)	0 (0.0%)	111 (3.7%)	184 (19.5%)	357 (59.1%)	37 (4.6%)
Married or living in a union	37 (2.8%)	678 (21.7%)	5635 (98.9%)	4856 (95.3%)	987 (100.0%)	2859 (96.3%)	0 (0.0%)	191 (31.6%)	760 (95.4%)
Never married nor lived in a union	1265 (94.2%)	65 (2.1%)	0 (0.0%)	0 (0.0%)	0 (0.0%)	0 (0.0%)	764 (80.5%)	56 (9.3%)	0 (0.0%)
Location									
Rural	1136 (84.6%)	3021 (96.6%)	5238 (92.0%)	5093 (100.0%)	0 (0.0%)	1716 (57.8%)	391 (41.3%)	0 (0.0%)	775 (97.3%)
Urban	207 (15.4%)	106 (3.4%)	457 (8.0%)	0 (0.0%)	987 (100.0%)	1254 (42.2%)	557 (58.7%)	604 (100.0%)	22 (2.7%)
Household head									
Female	524 (39.1%)	3127 (100.0%)	250 (4.4%)	1045 (20.5%)	98 (9.9%)	228 (7.7%)	456 (48.1%)	503 (83.2%)	90 (11.3%)
Male	819 (60.6%)	0 (0.0%)	5445 (95.6%)	4048 (79.5%)	889 (90.1%)	2742 (92.3%)	492 (51.9%)	101 (16.8%)	707 (88.7%)
Ever tested for HIV									
Never tested	689 (51.3%)	208 (6.7%)	0 (0.0%)	165 (3.2%)	0 (0.0%)	58 (2.0%)	57 (6.1%)	35 (5.8%)	422 (52.9%)
Tested	654 (48.7%)	2919 (93.3%)	5695 (100.0%)	4928 (96.8%)	987 (100.0%)	2912 (98.1%)	891 (93.9%)	569 (94.2%)	375 (47.1%)
Employment status									
Not currently employed	819 (61.0%)	588 (18.8%)	1510 (26.5%)	2192 (43.0%)	987 (100.0%)	230 (7.7%)	498 (52.6%)	49 (8.1%)	367 (46.1%)
Currently employed	524 (39.0%)	2539 (81.2%)	4185 (73.5%)	2901 (57.0%)	0 (0.0%)	2740 (92.3%)	450 (47.5%)	555 (91.9%)	430 (53.9%)
Cluster total	1343 (6.2%)	3127 (14.5%)	5695 (26.4%)	5093 (23.6%)	987 (4.6%)	2970 (13.8%)	948 (4.4%)	604 (2.8%)	797 (3.7%)
HIV status									
Negative	407 (30.3%)	759 (24.3%)	1526 (26.8%)	1529 (30.0%)	260 (26.3%)	780 (26.3%)	262 (27.6%)	135 (22.4%)	231 (29.0%)
Positive	34 (2.5%)	204 (6.5%)	228 (4.0%)	133 (2.6%)	45 (4.5%)	103 (3.5%)	29 (3.1%)	80 (13.3%)	5 (0.6%)
Unknown	902 (67.2%)	2164 (69.2%)	3941 (69.2%)	3431 (67.4%)	682 (69.1%)	2087 (70.3%)	657 (69.3%)	389 (64.4%)	561 (70.4%)
HIV prevalence^c^	7.7%	21.2%	13.0%	8.0%	14.8%	11.7%	10.0%	37.2%	2.1%

^a^ Can read a whole sentence

^b^ Cannot read at all, or can read part of a sentence only

^c^ We defined the prevalence as the proportion of positive tests among the conclusive (positive and negative) tests results.

HIV testing uptake was relatively low in *Groups 1* and *9* (*Group 1,* 48.7%; *Group 9,* 47.1%) compared to other groups where the median HIV testing uptake was 96.8% (IQR 94.1% to 99.1%). *Group 9* had a low HIV prevalence (2.1%) and consisted mainly of older (46.5% over 35 years) women who lived in rural areas (97.3%) and had a low level of literacy (29.4%). These women generally lived in a union (95.4%) and their household was male‐headed (88.7%). In *Group 1*, the HIV prevalence was higher (7.7%). This group included literate (73.3%), adolescent (77.8% under 20 years) girls who lived in rural (84.6%) areas and had never been in a union (94.2%). HIV testing uptake was approximately two‐fold higher (96.8%) in *Group 4* than *Group 1* (48.7%), although their HIV prevalence were similar (*Group 4,* 8.0%; *Group 1*, 7.7%). *Group 4* was characterized by young (83.1% aged between 20 and 35), rural (100.0%) and married (95.3%) women.

**Figure 1:**
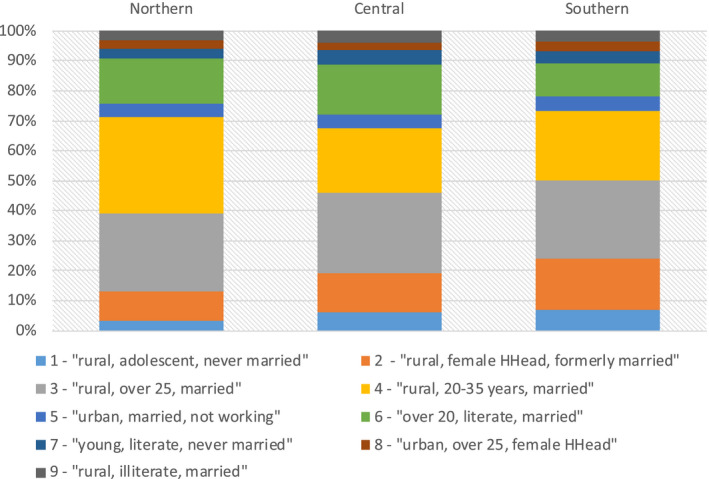
Proportion of the nine female groups in the three regions of Malawi: Northern, Central, and Southern

Figure 1 and Table S3 present the distribution of the nine groups of women in the Northern, Central and Southern regions of Malawi. The Southern region included a higher percentage (7.2%) of rural, literate, adolescent girls, who had never been in a union and who had a low uptake of HIV testing (*Group 1*); and a lower percentage (10.8%) of well tested married women aged over 20 (*Group 6*) than the Central (*Group 1*, 6.0%; *Group 6*, 16.7%) and Northern (*Group 1*, 3.3%; *Group 6*, 15.1%) regions. The Southern region had also a higher percentage (17.0%) of rural divorced/widowed women with a female household head and a high HIV prevalence (*Group 2*) than the other regions (Central, 13.1%; Northern, 9.6%). In contrast, the Northern region had a higher percentage (32.5%) of young rural married women (*Group 4*) with a high HIV testing uptake than the Central (21.6%) and Southern (23.2%) regions.

**Figure 2:**
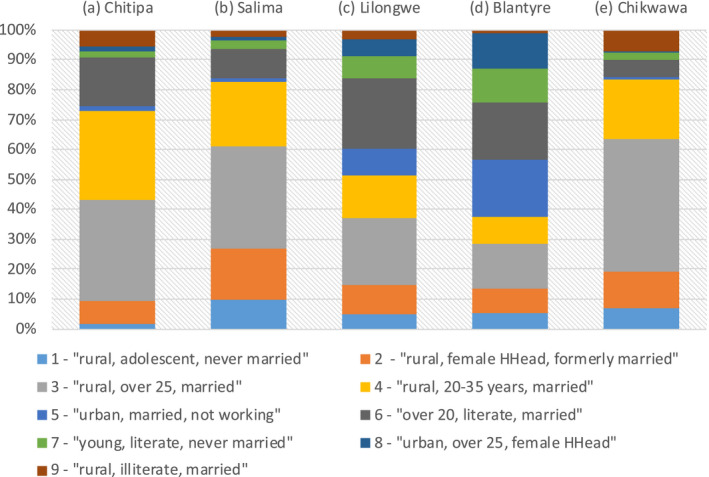
Proportion of the nine female groups in five selected districts of Malawi: (a) Chitipa, (b) Salima, (c) Lilongwe, (d) Blantyre, (e) Chikwawa

The distribution of female groups varied also across the 28 districts of Malawi (Table S4). Figure 2 presents the proportion of the nine groups in five selected districts that cover all three regions of the country: Chitipa (Northern), Salima (Central), Lilongwe (Central), Blantyre (Southern) and Chikwawa (Southern region). We selected two neighbouring districts from the same region (Salima and Lilongwe in the Central region; Chikwawa and Blantyre in the Southern region) because they had remarkably different HIV prevalence (Table S5 and Figure S1) (Salima, 5.2% vs. Lilongwe, 9.2%; Chikwawa, 9.1% vs. Blantyre, 24.1%). The proportion of female groups in high and low prevalence districts are available in Table S6.

### 3.2 Men’s groups

The optimal number of groups for men was six based on 11 variables: age, age at first sex, marital status, location of residence, having ever been tested for HIV, media access, employment, literacy, sex of household head, wife beating justification and willingness to use condom (Table 2). Entropy statistic and average posterior probabilities from LCA for men are provided in Table S7. Compared to women, men had a lower HIV prevalence (8.4% vs 12.7%); and a lower uptake of HIV testing. The percentage of men who had never been tested for HIV was three times higher than for women (22.5% vs. 7.6%). Two groups of men, *Groups 3* and *5,* had a particularly low testing uptake (52.5% and 35.0%; vs. 80.5% to 92.2% in other groups). Although literacy was substantially different between these groups (*Group 3,* 97.7%; *Group 5,* 29.7%), men in both groups had many similar characteristics: they were mainly adolescents (77.7% under 20 years in *Group 3*; 70.3% in *Group 5*), had never lived in a union (*Group 3,* 96.0%; *Group 5,* 98.2%), and lived in rural settings (*Group 3,* 80.3%; *Group 5,* 97.2%). These groups also had the lowest HIV prevalence (0.8% and 0.5%).

**Table 2:** Groups of men based on the 11 most important variables: age, age at first sex, marital status, location of residence, having ever been tested for HIV, media access, employment, literacy, sex of household head, wife beating justification and willingness to use condom. Data from 6379 men were analysed

Group	1	2	3	4	5	6
Age						
19 and under	19 (1.0%)	0 (0.0%)	570 (77.7%)	0 (0.0%)	314 (70.3%)	12 (2.7%)
20 to 25	321 (16.1%)	535 (72.7%)	164 (22.3%)	3 (0.1%)	125 (28.0%)	215 (47.8%)
26 to 35	689 (34.7%)	167 (22.7%)	0 (0.0%)	827 (40.9%)	3 (0.7%)	217 (48.3%)
Over 35	960 (48.3%)	34 (4.6%)	0 (0.0%)	1193 (59.0%)	5 (1.1%)	6 (1.2%)
Age at first sex						
Under 16	713 (35.9%)	158 (21.4%)	479 (65.3%)	355 (17.5%)	294 (65.8%)	58 (12.8%)
16 to 18	393 (19.8%)	143 (19.4%)	194 (26.4%)	296 (14.7%)	106 (23.8%)	122 (27.1%)
19 to 21	608 (30.6%)	339 (46.0%)	61 (8.3%)	692 (34.2%)	47 (10.4%)	225 (50.1%)
Over 21	275 (13.8%)	97 (13.2%)	0 (0.0%)	680 (33.6%)	0 (0.0%)	45 (10.0%)
Current marital status						
Formerly married or lived in a union	132 (6.7%)	73 (9.9%)	0 (0.0%)	49 (2.4%)	2 (0.4%)	0 (0.0%)
Married or living in a union	1828 (92.0 %)	52 (7.1%)	30 (4.0%)	1974 (97.6%)	7 (1.5%)	450 (100.0%)
Never married nor lived in a union	27 (1.4%)	612 (83.1%)	704 (96.0%)	1 (0.0%)	439 (98.2%)	0 (0.0%)
Location						
Rural	1925 (96.8%)	385 (52.3%)	589 (80.3%)	1509 (74.6%)	435 (97.2%)	357 (79.3%)
Urban	63 (3.2%)	352 (47.7%)	145 (19.7%)	514 (25.4%)	12 (2.8%)	93 (20.7%)
Ever tested for HIV						
Never tested	387 (19.5%)	101 (13.7%)	348 (47.5%)	272 (13.4%)	290 (65.0%)	35 (7.8%)
Tested	1601 (80.5%)	636 (86.3%)	386 (52.5%)	1751 (86.6%)	157 (35.0%)	415 (92.2%)
Has media access						
No	1333 (67.05%)	201 (27.28%)	306 (41.74%)	518 (25.63%)	283 (63.26%)	103 (22.85%)
Yes	655 (33.0%)	536 (72.7%)	428 (58.3%)	1505 (74.4%)	164 (36.4%)	347 (77.2%)
Employment status						
Not currently employed	185 (9.3%)	143 (19.3%)	303 (41.2%)	30 (1.5%)	128 (28.7%)	0 (0.0%)
Currently employed	1803 (90.7%)	594 (80.7%)	431 (58.8%)	1993 (98.5%)	319 (71.3%)	450 (100.0%)
Literacy						
Good reader^a^	863 (43.4%)	642 (87.1%)	717 (97.7%)	1777 (87.9%)	133 (29.7%)	440 (97.8%)
Poor reader^b^	1125 (56.6%)	95 (12.9%)	17 (2.3%)	246 (12.2%)	314 (70.3%)	10 (2.2%)
Household head						
Female	127 (6.4%)	212 (28.8%)	270 (36.7%)	48 (2.4%)	120 (26.8%)	32 (7.1%)
Male	1861 (93.6%)	525 (71.2%)	464 (63.3%)	1975 (97.6%)	327 (73.2%)	418 (92.9%)
Beating wife justified						
No	1753 (88.2%)	645 (87.6%)	578 (78.8%)	1972 (97.5%)	308 (68.9%)	345 (76.7%)
Yes	235 (11.8%)	92 (12.4%)	156 (21.3%)	51 (2.5%)	139 (31.1%)	105 (23.4%)
Wife justified asking husband to use condom if he has STI^c^						
No	365 (18.4%)	45 (6.2%)	51 (6.9%)	38 (1.9%)	107 (24.0%)	45 (10.1%)
Yes	1623 (81.6%)	692 (93.9%)	683 (93.1%)	1985 (98.1%)	340 (76.0%)	405 (89.9%)
Cluster total	1988 (31.2%)	737 (11.6%)	734 (11.5%)	2023 (31.7%)	447 (7.0%)	450 (7.1%)
HIV status						
Negative	1573 (79.1%)	613 (83.2%)	652 (88.8%)	1568 (77.5%)	396 (88.6%)	366 (81.3%)
Positive	175 (8.8%)	42 (5.7%)	5 (0.7%)	235 (11.6%)	2 (0.5%)	15 (3.3%)
Unknown	240 (12.1%)	82 (11.1%)	77 (10.5%)	220 (10.9%)	49 (11.0%)	69 (15.3%)
HIV prevalence^d^	10.0%	6.4%	0.8%	13.0%	0.5%	3.9%

^a^ Can read a whole sentence

^b^ Cannot read at all, or can read part of a sentence only

^c^ Sexually Transmitted Infection

^d^ We defined the prevalence as the proportion of positive tests among the conclusive (positive and negative) tests results.

The groups of men with the highest HIV prevalence were *Groups 1* and *4* (*Group 1,* 10.0%*; Group 4,* 13.0%). These groups represented 31.2% and 31.7% of all men, respectively, and had a relatively high HIV testing uptake (*Group 1*, 80.5%; *Group 4,* 86.6%). They both had a high proportion of older men (48.3% aged over 35 in *Group 1;* 59.0% in *Group 4*), who were mainly living in a union (*Group 1,* 92.0%*; Group 4,* 97.6%). The household heads were also mostly men (*Group 1,* 93.6%*; Group 4*, 97.6%). In contrast, *Group 4* was more urban than *Group 1* (25.4% vs. 3.2%), and access to media in it was better (74.4% vs. 33.0%). The group with the third highest HIV prevalence was *Group 2* (6.4%). This group had a high proportion of young men (72.7% between 20 and 25 years old), who were literate (87.1%) and had never lived in a union (83.1%). In comparison to *Group 2*, *Group 6*, which included mainly men who were literate (97.8%), aged between 20 and 35 (96.1%), had regular access to media (77.2%), and were living in a union (100.0%) and in rural settings (79.3%), had a lower HIV prevalence (3.9%) and a higher HIV testing uptake (*Group 6,* 92.2%; *Group 2,* 86.3%).

**Figure 3:**
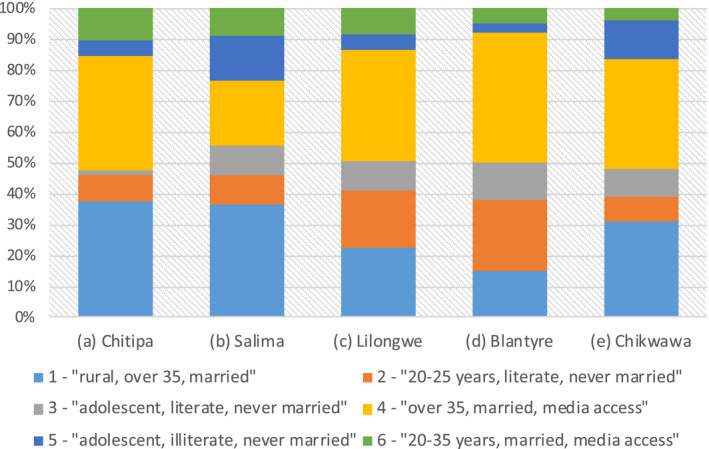
Proportion of the six male groups in five selected districts of Malawi. (a) Chitipa, (b) Salima, (c) Lilongwe, (d) Blantyre, (e) Chikwawa

Figure 3 presents the proportion of the six groups in the districts of Chitipa (Northern), Salima (Central), Lilongwe (Central), Blantyre (Southern) and Chikwawa (Southern region). In both Central and Southern regions, the districts with higher HIV prevalence (Lilongwe; Blantyre) were characterized by a lower proportion of older men (over 35) living in rural areas, who were in a union and did not have regular access to media (22.8% of *Group 1* in Lilongwe vs. 36.5% in Salima; 15.0% in Blantyre vs. 30.7% in Chikwawa). Lilongwe and Blantyre also had a lower percentage of rural, illiterate adolescents with a low HIV prevalence (4.9% of *Group 5* in Lilongwe; 3.3% in Blantyre) than Salima and Chikwawa (14.5% of *Group 5* in Salima; 12.2% in Chikwawa). Higher HIV prevalence coincided also with a higher percentage of young, literate men, who had never lived in a union (18.5% of *Group 2* in Lilongwe vs. 9.4% in Salima; 23.0% in Blantyre vs. 7.8 % in Chikwawa), and a higher proportion of older (over 35) men, who were living in a union and had regular access to media (36.3% of *Group 4* in Lilongwe vs. 21.0% in Salima; 42.0% in Blantyre vs. 35.1% in Chikwawa). Both districts of the Southern region (Blantyre and Chikwawa) were characterized by a lower proportion of literate, married men, who were aged 20 to 35 and lived in rural settings (4.7% of *Group 6* in Blantyre; and 4.9% in Chikwawa vs. 10.2%), compared to Chitipa (Northern region). In contrast, Chitipa was characterized by a lower percentage of rural, illiterate, adolescent men (*Group 6,* 5.1%) than the rural districts of Southern and Central regions (Chikwawa, 12.2%; Salima, 14.5%). The proportion of the six male groups in the three regions, 28 districts, and in high and low prevalence districts of Malawi are provided in Tables S8‐S10 and Figure S2.

## 4 DISCUSSION

Using LCA, we categorized Malawian women into nine groups and men into six groups with common sociobehavioural characteristics. Among these groups, we identified profiles of women and men who were most at risk of being HIV positive and less tested for HIV. Female household heads bore the heaviest burden of HIV, and rural adolescent girls had a significantly lower HIV testing uptake despite a relatively high HIV prevalence. Among men, older ones were the most affected by HIV, followed by young men who had never been married. Mapping the identified groups across districts of Malawi showed that the most vulnerable groups to HIV (higher prevalence and lower testing) were more frequent in the Southern region and urban districts of the country.

The groups of women with the highest HIV prevalence were characterized by an older age (over 25 years), living in a female‐headed household and having lived formerly in a union. This finding is consistent with earlier studies that showed that women who were no longer in a union were most‐at‐risk of being HIV positive in Malawi [5]. The most common group of female household heads in Malawi are widows [21], and it is well possible that their husband died of AIDS. Indeed, the proportion of widows and orphans increased over time in the most HIV‐affected countries [22]. Female household heads were also shown to live more frequently in poverty, requiring support from dedicated programmes [21, 23].

The uptake of HIV testing in the identified groups was generally consistent with the risk of being HIV positive, with more persons being tested for HIV when HIV prevalence was high. Adolescents (<20 years) were tested much less frequently than adults [24, 25, 26]. The low HIV testing uptake among adolescents is concerning, because the proportion of young people (15 to 24) among all AIDS‐related deaths in Malawi increased from 3.1% to 11.5% between 2004 and 2018 [1]. In particular, rural adolescent girls, who have never lived in a union, were tested about half as much as older (20 to 35 years), rural, literate women, who were living in a union, and had a similar relatively high HIV prevalence. Yet, adolescent girls are a vulnerable group that often lack knowledge about HIV [24], tend to perceive themselves as being at low risk of HIV [27], and are prime targets of gender‐based violence [28]. These observations confirm the need to accelerate tailored HIV prevention programming among adolescents in general, and specifically among rural adolescent girls [29].

Men tended to have a lower HIV prevalence than women, confirming the gender disparity in HIV infection in SSA [30, 31]. Men were also less often tested for HIV, which could be influenced by harmful masculinity stereotypes and cultural norms that inhibit men from seeking care [32, 33]. It may also result from the focus of HIV prevention in recent years on women and girls, and HIV testing being routinely performed in antenatal care. UNAIDS’ report released in 2017 [34] highlighted the low utilisation of health services by men and qualified it as a *blind spot* in the response to HIV. Men have therefore a higher risk of HIV transmission to their partners, and eventually a higher mortality due to AIDS‐related illnesses, affecting their families.

HIV prevalence was associated with the age and partnership status of men across the groups. Older men (>35 years) had the highest HIV prevalence regardless of their place of residence, and literacy. Compared to 2016, HIV incidence in Malawi was nearly three times higher in year 2000 [1], which can explain the higher risk of HIV acquisition among men who were young adults at that time. The current lower willingness to condom utilisation among rural, less educated, older and married men [35, 36] may also contribute to higher risk of recent HIV acquisition. Among young men, living in a union and in a rural setting was associated with a reduced risk of HIV. This finding is in accordance with a recent study performed in South Africa, where single never married men had a high risk of being HIV positive, which may be associated with a higher frequency of less stable relations and multiple sexual partners [37].

Groups of women and men with high HIV prevalence and low testing coverage were more frequent in urban areas of Malawi. Palk and Blower [8] showed that urban areas of Malawi also had a higher density of individuals with a high number of lifetime sexual partners. Women and men in the mostly rural Northern region were generally well tested. Our finding agrees with a recent study, which also reported a regional variance of HIV testing uptake in Malawi, with a better testing uptake in the North, although this study focused on men [25].

Our work has several strengths and limitations. Rather than analysing the association of single variables, we identified groups of patients with similar sociobehavioural characteristics. Our approach is therefore more closely related to general intuition and reasoning in the population than an analysis of individual risk factors. The identified groups are not directly observable using a single variable approach. The analysis was based on a limited number of factors that were selected by the researchers based on prior knowledge from the available literature. For this reason, some potentially important variables such as ethnicity or risky sexual behaviour had to be excluded. In order to avoid the subjective selection of the variables in the future, we are working in parallel on an automatic method to select the most important variables to predict individual HIV status. Our preliminary results showed that most of the variables that we selected in this study were highly relevant to predict HIV [38]. Our analysis allowed us to identify groups of men and women with associated probabilities of being HIV positive and tested for HIV, but the cross‐sectional nature of our analysis does not allow us to draw causal relations. There are other methods, such as Bayesian network analyses, that attempt to determine the possible causal pathways between various risk factors and HIV [39]. The data we used are also potentially biased because they are self‐reported.

## 5 CONCLUSIONS

Our findings can help focusing on evidence‐based interventions for maximum impact and improve targeting of critical interventions to key and vulnerable population as advocated in the National Strategic Plan for HIV and AIDS [40]. Further research should try to get a better understanding of vulnerable sub‐groups in different areas of SSA. Using LCA to identify and map vulnerable groups of women and men can provide key information to policy makers, enabling them to strategically design tailored support and prevention programmes that target people who are most in need. Our results clearly demonstrated that rural adolescent girls, young single men, and married men aged over 35 years have a particularly high risk of being HIV positive. Efforts should be made to increase the coverage of HIV testing in particular among these sub‐populations.

## COMPETING INTERESTS

We declare no competing interest.

## AUTHOR’S CONTRIBUTION

AM, JE, ZB and OK designed the study. AM and OK reviewed and selected the variables to be included in the analysis. AS wrote the code and performed the analysis with support from AM, ZB and KP. AM and AS interpreted the results with support from ZB, JE and OK. AM, AS, JE and OK reviewed the literature. AM wrote the manuscript, which was reviewed by AS, JE, ZB, KP and OK. All authors have read and approved the final manuscript. EO, OK and JE responded to reviewers’ comments.

## Supporting information


**Figure S1.** Prevalence of HIV by district for (left) women, and (right) men. Prevalence was defined as the proportion of positive HIV tests among all conclusive test results. Districts of (a) Chitipa, (b) Salima, (c) Lilongwe, (d) Blantyre and (e) Chikwawa are annotated on the map.

**Figure S2.** Distribution (%) of the six male groups in the three regions of Malawi: Northern, Central, and Southern.

**Table S1.** DHS datasets
**Table** **S2.** Entropy statistic and average posterior probabilities from LCA for women
**Table** **S3.** Distribution (%) of female groups per region: Southern, Central and Northern
**Table** **S4.** Distribution (%) of female groups per district
**Table** **S5.** HIV prevalence (%) of women and men per district in the DHS dataset. Prevalence was defined as the proportion of positive HIV tests among all conclusive test results
**Table** **S6.** Groups of women based on high and low prevalence districts
**Table** **S7.** Entropy statistic and average posterior probabilities from LCA for men
**Table** **S8.** Distribution of male groups per region: Southern, Central and Northern
**Table** **S9.** Distribution (%) of male groups per district
**Table** **S10.** Groups of men based on high and low prevalence districts
