# Supplementary figures and images for: Identifying groups of people with similar sociobehavioural characteristics in Malawi to inform HIV interventions: a latent class analysis
Source: J Int AIDS Soc. 2020 Sep 28;23(9):e25615. doi: 10.1002/jia2.25615 (PMC7521110; doi:10.1002/jia2.25615)

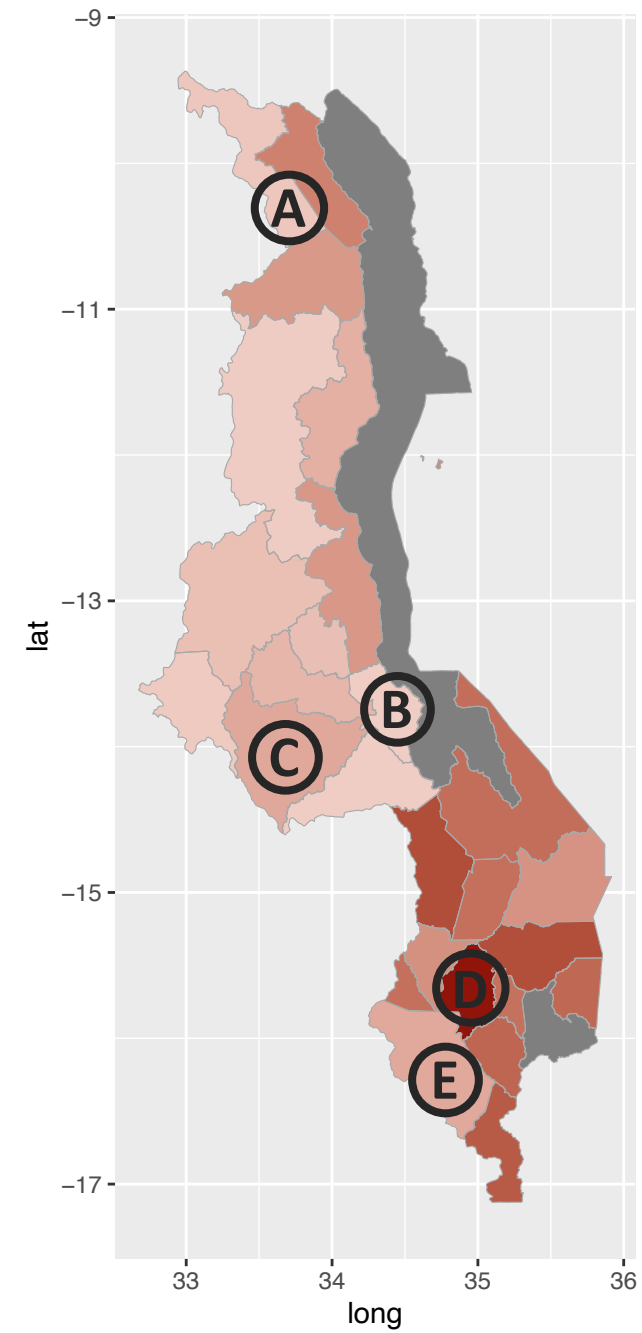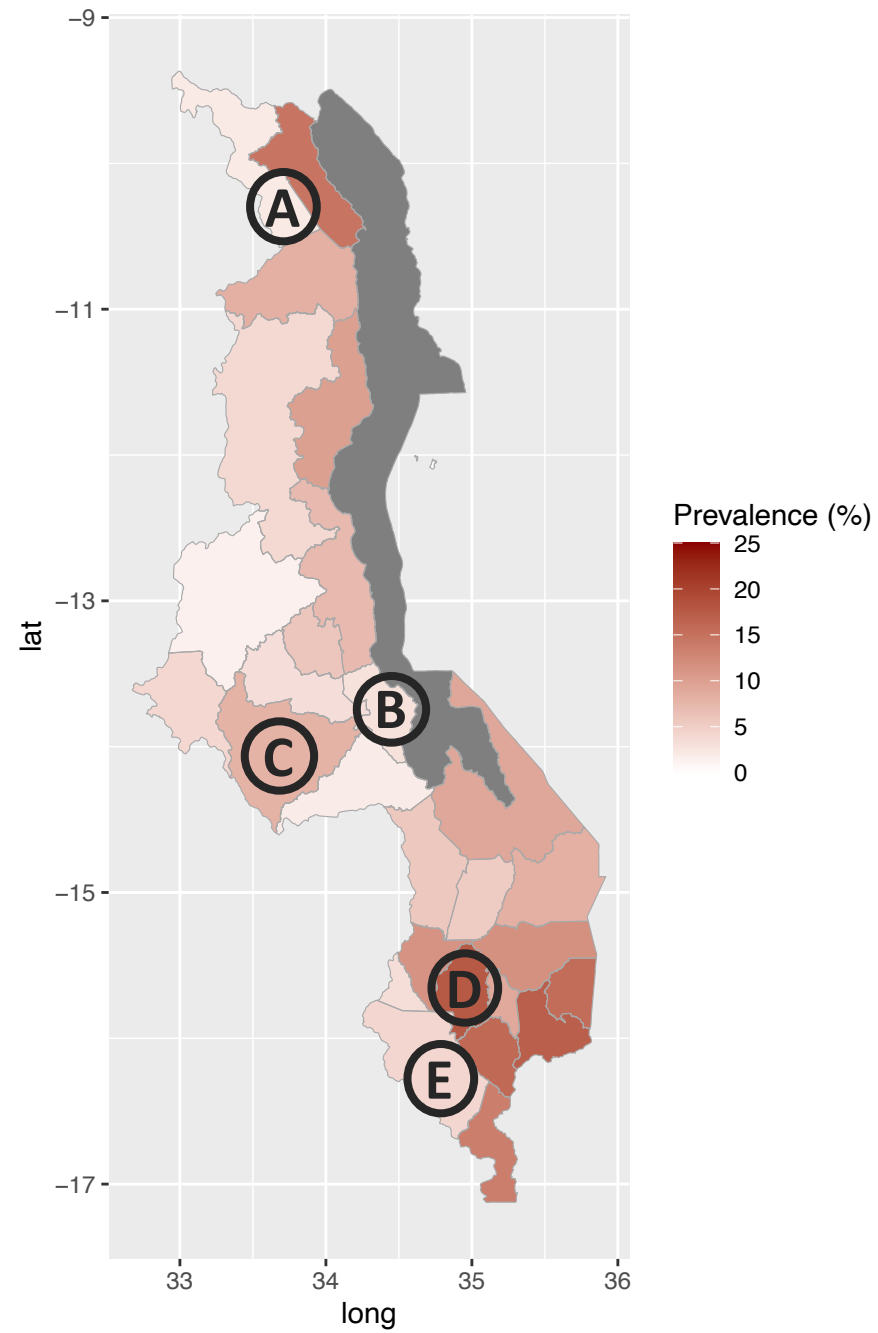

Supplement: Supplementary file 1 — Figure S1. Prevalence of HIV by district for (left) women, and (right) men. Prevalence was defined as the proportion of positive HIV tests among all conclusive test results. Districts of (a) Chitipa, (b) Salima, (c) Lilongwe, (d) Blantyre and (e) Chikwawa are annotated on the map. [file JIA2-23-e25615-s001.pdf]
