# Supplementary material for: Identifying groups of people with similar sociobehavioural characteristics in Malawi to inform HIV interventions: a latent class analysis
Source: J Int AIDS Soc. 2020 Sep 28;23(9):e25615. doi: 10.1002/jia2.25615 (PMC7521110; doi:10.1002/jia2.25615)
Supplement: Supplementary file 2 — Figure S2. Distribution (%) of the six male groups in the three regions of Malawi: Northern, Central, and Southern. [file JIA2-23-e25615-s002.pdf]

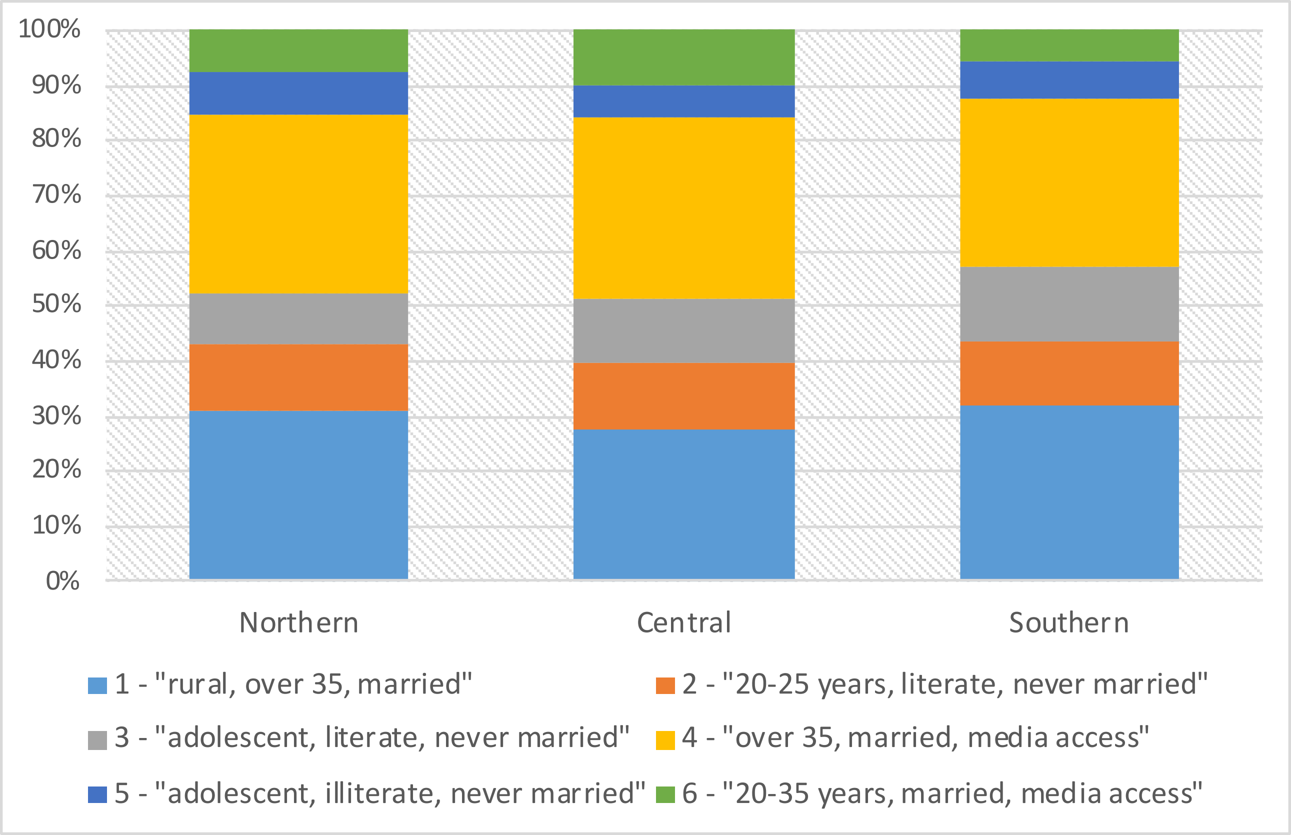
100%
90%
80%
70%
60%
50%
40%
30%
20%
10%
0%
Northern
Central
Southern
1 - "rural, over 35, married"
2 - "20-25 years, literate, never married"
3 - "adolescent, literate, never married"
4 - "over 35, married, media access"
5 - "adolescent, illiterate, never married"
6 - "20-35 years, married, media access"
