# Supplementary material for: Identifying groups of people with similar sociobehavioural characteristics in Malawi to inform HIV interventions: a latent class analysis
Source: J Int AIDS Soc. 2020 Sep 28;23(9):e25615. doi: 10.1002/jia2.25615 (PMC7521110; doi:10.1002/jia2.25615)
Supplement: Supplementary file 3 — Table S1. DHS datasets Table S2. Entropy statistic and average posterior probabilities from LCA for women Table S3. Distribution (%) of female groups per region: Southern, Central and Northern Table S4. Distribution (%) of female groups per district Table S5. HIV prevalence (%) of women and men per district in the DHS dataset. Prevalence was defined as the proportion of positive HIV tests among all conclusive test results Table S6. Groups of women based on high and low prevalence districts Table S7. Entropy statistic and average posterior probabilities from LCA for men Table S8. Distribution of male groups per region: Southern, Central and Northern Table S9. Distribution (%) of male groups per district Table S10. Groups of men based on high and low prevalence districts [file JIA2-23-e25615-s003.docx]

# Supplementary Material

## Data

We used three DHS datasets in this analysis (**Table S1**). We merged the HIV dataset with the male and female datasets separately, using a left outer join and the respondent datasets as the primary file. Respondents were indexed in male, female and HIV datasets by the columns: *DHS cluster number*, *household number* and *respondent’s line number*, which allowed us to merge the datasets.

**Table S1 - DHS Datasets**

| **File Name** | **Description** |
| --- | --- |
| MWMR7HDT.dat | Responses of the male respondents. |
| MWIR7HDT.dat | Responses of the female respondents. |
| MWAR7ADT.dat | HIV status of respondents (for a subset of male and female respondents). |

## HIV testing

HIV testing is simple; blood spots are collected on filter paper from a finger prick and transported to a laboratory for testing. The laboratory protocol includes an initial ELISA test, and then retesting of all positive tests and 5-10 percent of the negative tests with a second ELISA. For those with discordant results on the two ELISA tests, a new ELISA or a Western Blot is performed.

## Multiple Imputation

We imputed missing data on *age at first sex* and *literacy* using the following explanatory variables: place of residence, age, regular access to media, being currently employed, relationship status, sex of household head, justification of condom use when husband has a sexually transmitted infection (STI), justification of wife beating, comprehensive correct knowledge about AIDS, and HIV testing. In addition, the predictors included the following variables from the DHS: district of residence, religion, and accepting attitude toward people living with HIV/AIDS. The imputation was run five times, using the Predictive Mean Matching (PMM) method.

We imputed *literacy* when the interviewer had no card with the required language (10 women; 6 men) to test the ability of respondents to read, and when respondents were blind or visually impaired (30 women; 11 men). We imputed the *age at first sex* of respondents when it was lower than 10 years old or when their answer was “inconsistent” (779 women; 116 men).

## Variable selection/elimination

We started LCA with all 12 variables and used a backward elimination procedure. Iteratively, we computed for each variable the sum of variances of conditional category probabilities by class (group), removed the variable with lowest total variance and repeated the analysis using the remaining variables. We iterated at least five times and until the optimal number of classes became smaller than ten. We also ran the analyses keeping other combinations of the last few excluded variables than in the main iterations. Finally, we considered the analyses including the highest number of variables for each number of classes identified. We selected the analysis with a maximum number of variables, excluding however variables with total variance lower than a heuristically defined threshold (<0.005).

## Detailed results

**Table S2 – Entropy statistic and average posterior probabilities from LCA for women**

| **Group** | **1** | **2** | **3** | **4** | **5** | **6** | **7** | **8** | **9** |
| --- | --- | --- | --- | --- | --- | --- | --- | --- | --- |
| Sample entropy statistic: 4.4 | | | | | | | | | |
| Average posterior probabilities | 6.8% (0.3%) | 14.1% (0.7%) | 24.7%  (2.1%) | 23.4% (2.0%) | 3.7% (0.3%) | 13.8% (2.0%) | 4.3% (0.3%) | 2.3%  (0.2%) | 6.8% (1.5%) |

*In parenthesis: standard error*

**Table S3 – Distribution (%) of female groups per region: Southern, Central and Northern.**

| **Group** | **1** | **2** | **3** | **4** | **5** | **6** | **7** | **8** | **9** |
| --- | --- | --- | --- | --- | --- | --- | --- | --- | --- |
| **Southern** | 7.2 | 17.0 | 26.1 | 23.2 | 4.9 | 10.8 | 4.3 | 2.9 | 3.7 |
| **Central** | 6.0 | 13.1 | 26.9 | 21.6 | 4.3 | 16.7 | 4.9 | 2.7 | 3.8 |
| **Northern** | 3.3 | 9.6 | 26.0 | 32.5 | 4.4 | 15.1 | 3.2 | 2.6 | 3.3 |

**Table S4 – Distribution (%) of female groups per district.**

| **Groups** | **1** | **2** | **3** | **4** | **5** | **6** | **7** | **8** | **9** |
| --- | --- | --- | --- | --- | --- | --- | --- | --- | --- |
| **Central** |  |  |  |  |  |  |  |  |  |
| Dedza | 5.9 | 24.2 | 27.0 | 23.7 | 0.2 | 10.7 | 2.3 | 0.7 | 5.3 |
| Dowa | 4.5 | 13.1 | 32.0 | 24.0 | 1.7 | 12.9 | 2.4 | 0.8 | 8.6 |
| Kasungu | 5.2 | 9.3 | 30.7 | 26.6 | 2.1 | 17.7 | 4.1 | 1.0 | 3.3 |
| Lilongwe | 5.2 | 9.5 | 22.2 | 14.6 | 8.9 | 23.4 | 7.5 | 5.6 | 3.1 |
| Mchinji | 8.5 | 15.6 | 28.7 | 25.1 | 1.4 | 15.9 | 2.7 | 0.3 | 1.7 |
| Nkhotakota | 7.6 | 13.0 | 33.8 | 26.6 | 1.4 | 9.0 | 2.8 | 1.4 | 4.4 |
| Ntcheu | 8.1 | 17.2 | 23.9 | 30.8 | 1.1 | 10.3 | 4.9 | 0.6 | 3.2 |
| Ntchisi | 1.9 | 11.6 | 34.2 | 36.4 | 0.8 | 9.1 | 1.9 | 0.3 | 3.9 |
| Salima | 10.0 | 16.9 | 34.2 | 21.5 | 1.3 | 9.6 | 3.1 | 1.1 | 2.4 |
| **Northern** |  |  |  |  |  |  |  |  |  |
| Chitipa | 1.7 | 7.8 | 33.9 | 29.4 | 1.7 | 16.1 | 2.2 | 1.7 | 5.6 |
| Karonga | 2.8 | 12.0 | 31.9 | 26.8 | 4.0 | 12.0 | 3.8 | 3.8 | 3.1 |
| Likoma | 11.1 | 11.1 | 22.2 | 27.8 | 0.0 | 16.7 | 5.6 | 5.6 | 0.0 |
| Mzimba | 2.9 | 8.2 | 24.3 | 33.8 | 6.3 | 14.5 | 3.8 | 2.7 | 3.6 |
| Nkhata Bay | 5.2 | 12.9 | 26.2 | 37.2 | 1.0 | 13.3 | 1.3 | 0.7 | 2.3 |
| Rumphi | 4.8 | 10.3 | 19.9 | 32.0 | 2.2 | 24.3 | 1.8 | 2.9 | 1.8 |
| **Southern** |  |  |  |  |  |  |  |  |  |
| Balaka | 10.2 | 18.8 | 24.5 | 25.7 | 2.5 | 10.3 | 4.2 | 1.5 | 2.3 |
| Blantyre | 5.3 | 8.2 | 14.9 | 8.9 | 19.5 | 18.7 | 11.7 | 11.8 | 1.0 |
| Chikwawa | 7.0 | 12.2 | 44.2 | 19.9 | 0.8 | 5.9 | 2.3 | 0.5 | 7.2 |
| Chiradzulu | 7.7 | 19.5 | 26.5 | 32.1 | 0.2 | 8.1 | 4.0 | 0.2 | 1.6 |
| Machinga | 4.7 | 18.2 | 37.0 | 26.6 | 1.1 | 6.4 | 1.4 | 1.2 | 3.3 |
| Mangochi | 9.9 | 17.5 | 23.5 | 32.3 | 1.7 | 4.2 | 1.2 | 0.3 | 9.4 |
| Mulanje | 7.8 | 23.9 | 29.5 | 22.8 | 0.4 | 10.9 | 2.3 | 0.3 | 2.1 |
| Mwanza | 5.6 | 17.3 | 22.8 | 18.5 | 5.6 | 14.2 | 2.5 | 1.2 | 12.4 |
| Neno | 4.6 | 22.3 | 29.4 | 25.2 | 0.0 | 11.8 | 3.4 | 1.3 | 2.1 |
| Nsanje | 8.8 | 12.8 | 37.3 | 18.8 | 2.9 | 10.5 | 2.9 | 0.6 | 5.4 |
| Phalombe | 6.0 | 25.0 | 22.2 | 30.7 | 0.4 | 12.6 | 0.9 | 0.0 | 2.3 |
| Thyolo | 9.1 | 22.9 | 28.0 | 23.7 | 0.7 | 9.0 | 2.4 | 0.4 | 3.8 |
| Zomba | 5.7 | 15.9 | 24.6 | 26.3 | 4.4 | 13.0 | 5.4 | 3.6 | 1.2 |

**Table S5 – HIV prevalence (%) of women and men per district in the DHS dataset.** Prevalence was defined as the proportion of positive HIV tests among all conclusive test results.

| **District** | **Prevalence (Women)** | **Prevalence (Men)** |
| --- | --- | --- |
| Balaka | 15.2 | 5.3 |
| Blantyre | 24.1 | 17.7 |
| Chikwawa | 9.1 | 4.3 |
| Chiradzulu | 15.1 | 9.1 |
| Chitipa | 5.9 | 2.2 |
| Dedza | 5.3 | 2.1 |
| Dowa | 7.6 | 3.4 |
| Karonga | 13.4 | 14.7 |
| Kasungu | 6.7 | 1.6 |
| Likoma | 12.5 | 0.0 |
| Lilongwe | 9.2 | 8.0 |
| Machinga | 11.4 | 8.3 |
| Mangochi | 15.5 | 9.3 |
| Mchinji | 5.5 | 4.2 |
| Mulanje | 25.9 | 17.0 |
| Mwanza | 15.2 | 3.4 |
| Mzimba | 5.4 | 4.0 |
| Neno | 11.7 | 11.5 |
| Nkhata Bay | 8.2 | 10.0 |
| Nkhotakota | 11.0 | 7.2 |
| Nsanje | 17.6 | 13.7 |
| Ntcheu | 18.9 | 5.8 |
| Ntchisi | 6.8 | 6.0 |
| Phalombe | 16.1 | 15.6 |
| Rumphi | 10.8 | 8.3 |
| Salima | 5.2 | 3.2 |
| Thyolo | 16.3 | 16.0 |
| Zomba | 18.8 | 11.7 |

**Table S6 - Groups of women based on high and low prevalence districts**

High (low) prevalence for urban and rural districts were defined as districts with a higher (lower) prevalence rate than the country urban and rural prevalence rate

| **Group** | **1** | **2** | **3** | **4** | **5** | **6** | **7** | **8** | **9** |
| --- | --- | --- | --- | --- | --- | --- | --- | --- | --- |
| High prevalence - urban | 55(4.1%) | 19(0.6%) | 136(2.4%) | 0(0.0%) | 450(45.6%) | 401(13.5%) | 252(26.6%) | 290(48.0%) | 11(1.4%) |
| Low prevalence - urban | 80(6.0%) | 31(1.0%) | 137(2.4%) | 0(0.0%) | 537(54.4%) | 811(27.3%) | 384(40.5%) | 314(52.0%) | 9(1.1%) |
| High prevalence - rural | 649(48.3%) | 1609(51.5%) | 2105(37.0%) | 2253(44.2%) | 0(0.0%) | 666(22.4%) | 180(19.0%) | 0(0.0%) | 314(39.4%) |
| Low prevalence - rural | 559(41.6%) | 1468(46.9%) | 3317(58.2%) | 2840(55.8%) | 0(0.0%) | 1092(36.8%) | 132(13.9%) | 0(0.0%) | 463(58.1%) |

High prevalence urban districts: Balaka, Blantyre, Karonga, Machinga, Mwanza, Zomba

High prevalence rural districts: Balaka, Blantyre, Chiradzulu, Likoma, Mangochi, Mulanje, Mwanza, Neno, Nsanje, Ntcheu, Phalombe, Thyolo, Zomba

**Table S7 – Entropy statistic and average posterior probabilities from LCA for men**

| **Group** | **1** | **2** | **3** | **4** | **5** | **6** |
| --- | --- | --- | --- | --- | --- | --- |
| Sample entropy statistic: 6.1 | | | | | | |
| Average posterior probabilities | 31.2% (1.8%) | 11.5% (0.6%) | 10.5%  (1.2%) | 29.5% (2.4%) | 8.6% (1.2%) | 8.6% (1.8%) |

*In parenthesis: standard error*

**Table S8 - Distribution of male groups per region: Southern, Central and Northern.**

| **Group** | **1** | **2** | **3** | **4** | **5** | **6** |
| --- | --- | --- | --- | --- | --- | --- |
| **Southern** | 32.1% | 11.3% | 13.7% | 30.5% | 6.9% | 5.6% |
| **Central** | 27.8% | 11.9% | 11.4% | 33.2% | 5.8% | 9.9% |
| **Northern** | 31.2% | 11.7% | 9.5% | 32.5% | 7.5% | 7.6% |

**Table S9 - Distribution (%) of male groups per district.**

| **Groups** | **1** | **2** | **3** | **4** | **5** | **6** |
| --- | --- | --- | --- | --- | --- | --- |
| **Central** |  |  |  |  |  |  |
| Dedza | 40.9 | 5.1 | 11.0 | 24.8 | 12.0 | 6.2 |
| Dowa | 37.6 | 9.2 | 3.2 | 31.6 | 9.9 | 8.5 |
| Kasungu | 41.6 | 4.6 | 8.8 | 34.0 | 8.0 | 3.1 |
| Lilongwe | 22.8 | 18.5 | 9.1 | 36.3 | 4.9 | 8.5 |
| Mchinji | 36.9 | 7.3 | 12.5 | 28.8 | 8.2 | 6.4 |
| Nkhotakota | 26.1 | 8.7 | 24.2 | 34.8 | 2.5 | 3.7 |
| Ntcheu | 31.9 | 12.0 | 6.5 | 33.3 | 4.2 | 12.0 |
| Ntchisi | 33.6 | 4.0 | 4.8 | 36.8 | 12.8 | 8.0 |
| Salima | 36.5 | 9.4 | 9.8 | 21.0 | 14.5 | 8.9 |
| **Northern** |  |  |  |  |  |  |
| Chitipa | 37.3 | 8.5 | 1.7 | 37.3 | 5.1 | 10.2 |
| Karonga | 30.1 | 9.8 | 11.9 | 34.3 | 2.8 | 11.2 |
| Likoma | 28.6 | 0.0 | 28.6 | 42.9 | 0.0 | 0.0 |
| Mzimba | 27.1 | 13.5 | 10.6 | 31.3 | 6.9 | 10.6 |
| Nkhata Bay | 26.1 | 9.1 | 10.2 | 38.6 | 5.7 | 10.2 |
| Rumphi | 23.1 | 12.8 | 21.8 | 32.1 | 6.4 | 3.9 |
| **Southern** |  |  |  |  |  |  |
| Balaka | 37.8 | 9.8 | 13.3 | 25.2 | 7.0 | 7.0 |
| Blantyre | 15.0 | 23.0 | 12.0 | 42.0 | 3.3 | 4.7 |
| Chikwawa | 30.7 | 7.8 | 9.3 | 35.1 | 12.2 | 4.9 |
| Chiradzulu | 31.1 | 5.6 | 23.3 | 31.1 | 0.0 | 8.9 |
| Machinga | 42.6 | 7.1 | 8.3 | 29.0 | 9.5 | 3.6 |
| Mangochi | 34.0 | 10.6 | 16.2 | 18.9 | 15.0 | 5.3 |
| Mulanje | 36.2 | 7.9 | 16.9 | 33.1 | 3.2 | 2.8 |
| Mwanza | 39.4 | 9.1 | 9.1 | 27.3 | 12.1 | 3.0 |
| Neno | 37.7 | 4.9 | 11.5 | 29.5 | 11.5 | 4.9 |
| Nsanje | 37.2 | 6.4 | 24.4 | 25.6 | 5.1 | 1.3 |
| Phalombe | 52.7 | 3.8 | 11.8 | 21.0 | 5.4 | 5.4 |
| Thyolo | 29.9 | 11.6 | 18.3 | 32.0 | 2.1 | 6.0 |
| Zomba | 31.0 | 11.5 | 10.2 | 30.7 | 6.8 | 9.9 |

**Table S10 - Groups of men based on high and low prevalence districts**

High (low) prevalence for urban and rural districts were defined as districts with a higher (lower) prevalence rate than the country urban and rural prevalence rate

| **Group** | **1** | **2** | **3** | **4** | **5** | **6** |
| --- | --- | --- | --- | --- | --- | --- |
| High prevalence - urban | 16  (0.8%) | 129  (17.5%) | 61  (8.3%) | 214 (10.6%) | 2 (0.4%) | 23 (5.1%) |
| Low prevalence - urban | 25  (1.3%) | 227  (30.8%) | 60  (8.2%) | 316  (15.6%) | 10  (2.2%) | 70  (15.6%) |
| High prevalence - rural | 828  (41.6%) | 163  (22.1%) | 320 (43.6%) | 610  (30.2%) | 156  (34.9%) | 135  (30.0%) |
| Low prevalence - rural | 1119  (56.3%) | 218  (29.6%) | 293  (39.9%) | 883  (43.6%) | 279  (62.4%) | 222  (49.3%) |

High prevalence urban districts: Blantyre, Machinga, Mchinji, Mulanje, Nkhata Bay, Ntcheu, Rumphi, Thyolo, Zomba

High prevalence rural districts: Blantyre, Chiradzulu, Karonga, Machinga, Mangochi, Mulanje, Neno, Nkhata Bay, Nkhotakota, Nsanje, Phalombe, Thyolo, Zomba
